# Restoring the autonomic balance to reduce liver steatosis

**DOI:** 10.1113/JP278567

**Published:** 2019-08-21

**Authors:** Eveline Bruinstroop, Eric Fliers, Andries Kalsbeek

**Affiliations:** ^1^ Department of Cardiovascular & Metabolic Disorders (CVMD) Duke‐NUS Medical School Singapore; ^2^ Department of Endocrinology and Metabolism Amsterdam UMC University of Amsterdam Amsterdam The Netherlands; ^3^ Netherlands Institute for Neuroscience Hypothalamic Integration Mechanisms Amsterdam The Netherlands

**Keywords:** autonomic nervous system, lipid metabolism, liver, obesity

The liver plays an important role in the switching between carbohydrate and lipid fuel metabolism during feeding and fasting. Recent research has provided clear evidence for a role of the autonomic nervous system in liver lipid metabolism, with opposing functions of its two branches (Bruinstroop *et al*. 2014). In a reductionist fashion, the autonomic nervous system is often depicted with the sympathetic nervous system being essential in fight or flight responses and the parasympathetic nervous system being important during rest and digest. For example, sympathetic nerve endings present on the heart and sweat glands cause an increased heart rate and sweat production in stressful situations, whereas parasympathetic nerve endings to the endocrine pancreas support insulin release during a meal. As for the liver, the sympathetic branch is most active during fasting, when the animal needs to search for food and has to rely on lipids as the main fuel source. On the other hand, parasympathetic input has been shown to be important post‐prandially when nutrients are digested and have to be stored for later use. Together, nutrients, hormones and the autonomic nervous system are all signalling systems aiming to maintain homeostasis at a condition optimal for survival.

Could an imbalance of the autonomic nervous system be involved in the pathogenesis of the metabolic syndrome? Currently, the overall health of our species in the western world not longer depends on the search for food, with homeostasis instead depending on strategies to maintain homeostasis during caloric overload. Almost 10 years ago, Licht *et al*. (2010) showed that increased sympathetic activity and decreased parasympathetic activity of the cardiovascular system, rather than stress hormones, are associated with components of the metabolic syndrome such as elevated serum triglycerides and hyperglycaemia. We previously hypothesized that sympathetic over‐activity therefore contributes to liver manifestations of the metabolic syndrome (Bruinstroop *et al*. 2014) and showed that ablation of sympathetic liver innervation in a genetic rat model of obesity reduces dyslipidaemia. In this issue of the *Journal of Physiology*, Hurr *et al*. (2014) now show that mice on a high‐fat diet display higher baseline activity in the liver sympathetic nerves compared to mice on a normal chow diet. To our knowledge, they are the first to measure higher baseline sympathetic activity towards the liver in obesity, a challenging procedure for many reasons. First, the depth of anaesthesia must be chosen carefully to anaesthetize the animal without reducing all nerve activity. Moreover, nerve activity is very sensitive to experimental manipulation. Finally, it is straightforward to show changes in nerve activity after an intervention in one recording in the same animal but much more difficult to compare nerve activity between animals. By cutting the nerve distal to the recording, it was concluded that the increased activity was a result of increased efferent signalling from the brain to the liver. At a central level, this increased sympathetic activity is probably driven by an increased hypothalamic neuropeptide Y (NPY) expression. Indeed, high‐fat feeding increases hypothalamic NPY expression, with genetic obesity models being characterized by increased NPY mRNA and protein levels. Moreover, NPY neurons are activated during fasting and an intact sympathetic innervation is necessary to mediate the stimulatory effects of NPY on hepatic lipid production.

Hurr *et al*. (2014) hypothesized that sympathetic overactivity may be a key contributor to (diet‐induced) hepatic steatosis. To test this hypothesis, both pharmacological whole body and liver specific techniques were employed to reduce sympathetic activity and reduced levels of liver triglycerides were found after both procedures, at least in the short term (≤1 week). This confirmation of the beneficial effects of decreasing sympathetic output on hepatic steatosis raises the question of whether neuromodulation could be a possible treatment for metabolic liver disease.

Non‐alcoholic fatty liver disease (NAFLD) starts with simple steatosis as a result of an imbalance between the delivery and production of fat in the liver and its subsequent secretion and metabolism. As a result of steatosis, hepatic inflammation and fibrosis may occur. To be considered as a potential treatment, it is important that the reduction of steatosis continues over the long term and also prevents the progression towards non‐alcoholic steatohepatitis. Interestingly, other studies showed that parasympathetic liver nerve activity may inhibit liver inflammation and, accordingly, in the later stages of NAFLD, parasympathetic stimulation may be more effective than sympathetic inhibition (Nishio *et al*. 2017). Therefore, more studies are needed to consider neuromodulation as a treatment option. Nevertheless, because currently no registered treatment options are available for NAFLD, neuromodulation clearly poses an interesting treatment modality.

In the field of hypertension, renal denervation has been investigated as a possible treatment since the 1930s. Moreover, hepatic denervation approaches are being considered as a treatment for diabetes. However, neuromodulation of the autonomic nervous system is not a straightforward treatment. Vagal nerve stimulation was approved by the US Food and Drug Administration as a treatment for obesity after pre‐clinical work showing weight loss, although, in humans, the results are not uniform. In our opinion, this is because we are still scratching the surface with respect to understanding how the autonomic nervous system functions. Computational modelling has shown that the mode of stimulation applied for the treatment of obesity in humans was blocking activity rather than stimulating nerve activity, possibly causing the negative results (Pelot & Grill, 2018). Therefore, it is important to consider both sympathetic and parasympathetic, as well as efferent and afferent, stimulatory and inhibitory effects, on liver metabolism. Effects on blood flow, inflammation, fibrosis, carcinogenesis and bile metabolism should be studied using physiological, bioengineering, anatomical, metabolic and computational techniques before employment of any neuromodulatory treatment. Otherwise, the situation is like driving in an autonomous car without any idea of where it might take you.

## Additional information

### Competing interests

No competing interests declared.

### Author contributions

EB drafted the manuscript. EF and AK edited and revised the first draft. All authors have approved the final version of the manuscript submitted for publication.

### Funding

No funding was received.
